# COVID-19 Infections in Long-Term Care Facilities in Wuhan, China: Challenges and Lessons

**DOI:** 10.1093/geroni/igaa057.058

**Published:** 2020-12-16

**Authors:** Yi Cai, Jing Wang, Bei Wu

**Affiliations:** 1 Wuhan University, Wuhan, China; 2 Fudan University, Nantong, Shanghai, China; 3 New York University, New York, New York, United States

## Abstract

The impact of the COVID-19 infection on older residents and the direct frontline workers in long-term care (LTC) facilities in Wuhan, China deserves close attention. The fatality rate for older residents infected by the COVID-19 is among the highest in China Viral outbreaks are likely to occur in LTC facilities due to group-living arrangements, lack of precautionary measures, and older residents’ vulnerability to diseases. In this study, we aimed to explore different stakeholders’ experiences and challenges in the midst of the spread of the virus in LTC facilities. We conducted telephone interviews with four groups from two LTC facilities and two hospitals: twelve older residents (Four suspected cases and two infected), ten family members, four direct frontline workers (two infected), two nursing home managers, and four health care professionals working in the two hospitals that infected older residents were transferred to. We found that the gap in the transition of care quality between LTC facilities and local hospitals was widened during the COVID-19 outbreak. LTC facilities were slow to take precautionary measures and underprepared to handle the crisis after the infections occurred. The wellbeing of older residents was significantly impacted during the transition, particularly for those with dementia. Health care professionals in local hospitals were under tremendous stress providing treatments for older residents while ensuring their safety. There is an urgent need to improve transitional care and the capacity in preventing and handling this type of crisis for older residents in LTC.

